# The Effect on Selenium Concentrations of a Randomized Intervention with Fish and Mussels in a Population with Relatively Low Habitual Dietary Selenium Intake

**DOI:** 10.3390/nu7010608

**Published:** 2015-01-15

**Authors:** Malene Outzen, Anne Tjønneland, Erik H. Larsen, Klaus K. Andersen, Jane Christensen, Kim Overvad, Anja Olsen

**Affiliations:** 1 Danish Cancer Society Research Center, Diet, Genes, and Environment, Strandboulevarden 49, DK-2100 Copenhagen Ø, Denmark; E-Mails: annet@cancer.dk (A.T.); anja@cancer.dk (A.O.); 2 Division of Food Chemistry, National Food Institute, Technical University of Denmark, Mørkhøj Bygade 19, DK-2860 Søborg, Denmark; E-Mail: ehlar@food.dtu.dk; 3 Danish Cancer Society Research Center, Statistics, Bioinformatics and Registry, Strandboulevarden 49, DK-2100 Copenhagen Ø, Denmark; E-Mails: klaus@cancer.dk (K.K.A.); jane@cancer.dk (J.C.); 4 Section for Epidemiology, Department of Public Health, Aarhus University, Bartholins Allé 2, DK-8000 Aarhus C, Denmark; E-Mail: ko@ph.au.dk; 5 Department of Cardiology, Aalborg University Hospital, Hobrovej 18–22, DK-9100 Aalborg, Denmark

**Keywords:** fish, intervention, mussels, randomized, selenium, selenoprotein P, shellfish

## Abstract

Selenium status of the Danish population is below that assumed optimal for the suggested protective effects against chronic diseases, including certain cancers. Fish and shellfish are important dietary sources of selenium in Denmark. We investigated the effect of increased fish and mussel intake on selenium blood concentrations in a population with relatively low habitual dietary selenium intake. We randomly assigned 102 healthy men and women (all non-smokers) aged 48–76 years to an intervention group (*n* = 51) or a control group (*n* = 51). Intervention participants received 1000 g fish and mussels/week for 26 weeks (~50 μg selenium/day). Controls received no intervention. Non-fasting blood samples were taken and whole blood selenium was determined using inductively coupled plasma-mass spectrometry (ICP-MS), and plasma selenoprotein P (SelP) was determined by high performance liquid chromatography coupled to ICP-MS. All available observations were included in linear multiple regression analysis to evaluate the effect of the intervention. The difference in mean change for intervention compared with control persons was 14.9 ng/mL (95% CI: 10.2, 19.7) for whole blood selenium, and 7.0 ng/mL (95% CI: 3.1, 10.9) for plasma SelP (Weeks 0–26). Selenium concentrations were significantly increased after 26 weeks of intervention, albeit to a lower degree than expected.

## 1. Introduction

Selenium is an essential trace element with important metabolic functions in human health including antioxidative and anti-inflammatory functions [1,2]. The biological effects of selenium occur through its incorporation in selenoproteins, one of the most predominant being selenoprotein P (SelP) [1,3]. SelP is a selenium transport protein that is a possible marker of the functional selenium status [3]. SelP responds to dietary selenium intake [4], and the maximal concentration of SelP in humans is expected to be obtained at plasma selenium concentrations of 125 ng/mL, corresponding to an intake of approximately 100 μg/day [5].

Selenium is postulated to prevent chronic diseases including prostate, colorectal, and lung cancer [1,2], but the evidence from randomized controlled trials and observational studies in humans has not been convincing [6]. It has been suggested that the association between selenium and risk of cancer is nonlinear and, for some cancer types, U-shaped [1,2]. The risk of some cancer types may be reduced at serum or plasma concentrations between 120 and 160 ng/mL when compared with concentrations below 120 ng/mL [1,2]. The mean selenium status of the Danish population is 90–100 ng/mL [7,8] (corresponding to a dietary selenium intake of ~40–50 μg/day), which is below the level required to obtain the possible protective effects against cancer.

Exposure to selenium is mainly through intake of foods or dietary supplements [1]. Selenium is present in the soil and can enter the food chain through crops and animal feed [9,10]. In Europe, however, the soil concentration of selenium is low compared with other parts of the world, including parts of the United States [11,12]; consequently, plant-based foods in Europe are relatively low in selenium. Selenium supplementation to fertilizers has been done in Finland since 1985, illustrating that this is a feasible strategy for increasing dietary selenium intake and plasma selenium concentrations of an otherwise low-selenium population [13]. Selenium-rich food sources include fish and shellfish [2,11]. In regions with low soil selenium content, fish and shellfish are important selenium sources, but the bioavailability of selenium from these marine foods is relatively low (56%–88%) compared with other food items [14,15,16,17]. Furthermore, the selenium content in fish and shellfish varies, and high selenium contents are found in e.g. mussels, and lower contents are found in e.g. cod [18]. Other important dietary selenium sources in low-selenium geographic areas are meat products where the selenium content is influenced by the feed and mineral supplements consumed by the animals [1,11]. However, several clinical studies have shown that micronutrients given as supplements do not always result in the same health effects as micronutrients from foods [19]. This implies that intake of selenium through natural food sources may be preferred.

In Denmark, the selenium status at population levels is in the lower range. Fish is a potentially important source, and with the current mean intake of 130 g per week for adults [20], an increase in the intake of fish should be feasible. A higher intake of this selenium-rich food source may therefore increase the blood selenium concentrations in the Danish population. To our knowledge the effect of fish and shellfish intake on selenium status has been studied only in short-term intervention studies with few study participants [17,21]. These previous studies observed minor effects on blood selenium concentrations. Steady-state of selenium in plasma seems to be obtained after 26 weeks’ intervention according to a long-term supplementation study (200 μg selenium-enriched yeast/day) [22], which suggests that long-term dietary intervention studies are preferred to investigate the effect of fish and shellfish intake on selenium status in a population with low selenium concentrations.

The hypothesis of the present study was that an increased intake of fish and mussels would increase the blood concentrations of selenium and SelP in a population with relatively low habitual dietary selenium intake. Accordingly, we aimed to investigate the effect of increased fish and mussel intake on whole blood selenium and plasma SelP concentrations after 26 weeks of intervention.

## 2. Methods

### 2.1. Study Design

This study was a randomized dietary intervention study with two parallel groups.

The study was approved by the Danish Regional Committee on Biomedical Research Ethics for the Capital Region of Denmark (H-2-2010-033) in accordance with the Helsinki declaration. It was also approved by the Danish Data Protection Agency (2010-41-5111). All participants were given oral and written information about the study; written informed consent was obtained from all participants included in the study.

This study is registered at http://www.clinicaltrials.gov as DCS-53227244.

### 2.2. Study Population

The study took place in the northern part of Jutland, Denmark, from September 2010 to March 2011. Participants were recruited via local media, including newspaper advertisements. Eligibility for randomization was determined by potential participants completing a questionnaire three months before baseline measurements on lifestyle, diet, and health status.

We planned to recruit 100 men and women aged 50–74 years with a BMI of 18.5 kg/m^2^–28 kg/m^2^. The exclusion criteria included current smoking, intake of dietary supplements containing selenium three months before baseline measurements, frequent intake of fish and shellfish (>300 g/week), excessive intake of alcohol (according to the official Danish guidelines at study recruitment: women > 14 units of alcohol/week, men > 21 units of alcohol/week), strenuous exercise (>10 h/week), severe chronic disease, and frequent use of specified medication (including diabetic medicine, anticoagulant medicine, and medication for heart disease), or a cancer diagnosis within the past 5 years. Study participants were requested to inform the investigators of any changes regarding disease or medication occurring during the study.

### 2.3. Dietary Intervention

Participants in the intervention group were provided with 1000 g raw fish and raw or processed mussels (portion size of 200 g; five portions/week) once a week for 26 weeks. This amount corresponds to an intake of approximately 50.3 μg selenium/day (based on data from the Danish Food Composition Databank [18]). The participants received four or five different types of fish per week. Diversity in the type of fish provided was prioritized to ensure variation and thereby optimize compliance. In total, each participant received the following amounts during the entire intervention: Atlantic wolffish (0.2 kg, 115.0 μg selenium), salmon (farmed) (4.2 kg, 1352.4 μg selenium), herring (0.6 kg, 192.6 μg selenium), haddock (0.6 kg, 267.0 μg selenium), coalfish (2.8 kg, 907.2 μg selenium), cod (5.0 kg, 1435.0 μg selenium), plaice (5.2 kg, 1502.8 μg selenium), ling (0.6 kg, 254.4 μg selenium), and mussels (6.8 kg, 3128.0 μg selenium) [18]. In total, the estimated selenium intake from the intervention was 9154.4 μg/182 days. During the first eight weeks the intervention diet included 400 g blue mussels/week after which the amount was reduced to 200 g mussels/week. At study initiation, participants were instructed to consume the five portions of fish and mussels over a week, but there were no restrictions regarding the amount consumed per meal. The participants were allowed to consume other meals containing fish or shellfish besides the experimental foods.

At study start participants in the intervention group were given recipes for inspiration for meal preparation. To monitor their compliance they were provided with a self-monitoring record and kitchen scales to weigh the amount of received fish and mussels (prepared or raw) not consumed during the study period. At study initiation participants were instructed on how to complete the self-monitoring record.

### 2.4. Control Group

Participants in the control group received no intervention and were advised to maintain their habitual diets.

### 2.5. Data Collection

Non-fasting blood samples and anthropometric data were collected three times from each participant during the study: at baseline (September 2010) and after 13 weeks (December 2010) and 26 weeks (March 2011) of intervention. The blood samples were drawn from an antecubital vein with siliconized needles into K_2_-EDTA-coated blood drawing tubes of 6 mL (K_2_-EDTA BD Vacutainer Trace Elements REF 368381) and 10 mL (K_2_-EDTA BD Vacutainer REF 367525), respectively. The 6 mL blood samples were collected as whole blood. The 10 mL blood sample tubes were separated into plasma, erythrocytes, and buffy coat by centrifugation. For approximately every twenty-fifth blood sample collected, corresponding to one sample per day (performed by the same medical laboratory technicians), a field blank sample (MilliPore Super Q water, Millipore, Milford, MA, USA) was taken using the same equipment and was used as contamination control. All sample tubes were stored at room temperature for a maximum of eight hours from the time blood samples were drawn until they were stored in a freezer at −20 °C. The tubes were later stored in a freezer at −80 °C until analysis.

Anthropometric measurements (weight and height) were collected throughout the study by the same member of the professional staff. For all measurements, participants were without shoes and wore indoor clothing. BMI was calculated as weight (kg) divided by height squared (m^2^).

### 2.6. Power Analysis

Power analysis was done using a two-sample *t*-test and applying a significance level of 5% and a two-sided alternative when assuming comparison of changes from baseline to week 26 between the intervention and control group. It was determined that inclusion of 40 participants per group would allow a minimum detectable difference of 10 ng/mL (SD = 10) or 30 ng/mL (SD = 10) in selenium concentration between groups with a statistical power of 87% or 99%, respectively. The mean difference in the control group was assumed to be zero. To take into account a potential dropout of 20% in each group, we included 51 participants per group.

### 2.7. Randomization

Randomization was performed by a computer-generated procedure. The procedure ensured that the 24 couples included were randomly assigned to the same group—12 couples were randomly assigned to each group, and that men and women were equally distributed between the two groups—intervention group: 28 men and 23 women; control group: 29 men and 22 women.

### 2.8. Blinding

The laboratory staff that performed the biological and chemical analyses were blinded to the participants regarding the coupling between ID number and study group. The investigators were blinded to the ID numbers during the statistical analyses.

### 2.9. Chemical Analyses

Whole blood selenium analyses were conducted by an inductively coupled plasma-mass spectrometer equipped with a dynamic reaction cell, ICP-DRC-MS (ELAN 6100 DRC, Perkin-Elmer SCIEX, Concord, ON, Canada) to overcome interferences on the detection of selenium in accordance with a method described in detail in Nunes *et al.* [23]. Briefly, tetramethylammonium hydroxide was added to the thawed whole blood samples at a concentration of a 10% v/v. The samples were then incubated at room temperature for 10 min followed by dilution with a mixture of 0.05% w/v ethylenediamine tetraacetic acid and 0.005% v/v Triton X-100 surfactant to a 50-fold final dilution. Rhodium (^103^Rh) was used as internal standard, and the ratio of selenium as ^77^Se to ^103^Rh was measured using direct aspiration of the diluted blood samples into the ICP-MS instrument.

The concentration of SelP in plasma was determined using its selective retention by heparin-affinity high performance liquid chromatography (HPLC) and on-line detection by ICP-DRC-MS of selenium eluting from the HPLC column. Quantification was done by on-line post-column isotope dilution using a solution of enriched ^77^Se (99.3% pure) as spike isotope and ^80^Se as reference isotope for the natural selenium content of the biological samples [24]. The thawed plasma samples were centrifuged to separate the coagulum from the supernatant. The chromatographic system included an AF Pak AHR-894 heparin affinity column (8 mm × 50 mm; 18 μm particle size and 200 Å pore size) with a polyhydroxymethacrylate-based stationary phase (Showa Denko, Kawasaki, Japan). Mobile Phase A contained 20 mM ammonium nitrate (Sigma-Aldrich, MO, USA) and mobile Phase B an additional 500 units·mL^−1^ heparin, which is a highly sulfonated glycosaminoglycan (Wako Pure Chemical Industries Ltd., Osaka, Japan). In addition to determining SelP, the concentration of total selenium in plasma was also quantified by isotope dilution on the basis of the area under the complete chromatogram. The finding that the values obtained for total selenium did not deviate from the certified value of the BCR 637 Human Serum certified reference material (IRMM, Geel, Belgium) (target value *versus* obtained value) indicated that all the selenium contained in this plasma sample passed through the HPLC column and that the results were accurate. However, the method used to determine plasma selenium in this way was less precise than that used to determine selenium in whole blood. Therefore, the baseline plasma selenium concentration was used only for comparison with other Danish studies measuring plasma selenium in a healthy population.

All samples were analyzed by the same laboratory, and the same laboratory technicians performed the whole blood selenium and SelP analysis, respectively. For approximately every fifteenth unknown sample, the following samples were included for quality assurance of whole blood selenium and of SelP in plasma: one field blank to monitor possible contamination occurring during sample collection; one BCR 637 Human Serum CRM to monitor accuracy of the analyses of selenium in plasma or one whole blood laboratory check sample (ICP 07B03, Institut National de Santé Publique du Quebec, Quebec, Canada) to monitor the total selenium analyses in whole blood; and one duplicate sample to estimate analytical precision of total selenium or SelP across the complete analytical work. The derived analytical repeatability precision was 2.9% for whole blood selenium, 2.5% for plasma SelP, and 3.2% for plasma selenium. Furthermore, one laboratory blank sample was included in each batch of analyses for correction for random selenium contamination and for estimation of limit of detection (LOD). The LOD (3 × SD) was also estimated from the field blank sample, but this LOD was lower than that derived from the laboratory blank sample, and the latter was therefore used. The LOD values were 1.7 ng/mL for whole blood selenium, 6.3 ng/mL for SelP as selenium, and 4.9 ng/mL plasma.

### 2.10. Statistical Methods

The statistical analysis was based on all available observations. Participants who were randomized into a group, but did not attend the baseline appointment (Week 0) were excluded. Participants who attended only the baseline appointment were included only in the baseline characteristics.

Baseline characteristics on sex, age, BMI, whole blood selenium, plasma selenium, and plasma SelP are presented as medians with 5th and 95th percentiles for each study group.

Compliance with the intervention was estimated as follows: (total received amount—not ingested amount after preparation)/by total received amount.

Outlying observations were identified from visual inspection of the data (correlation plots of Week 0 *vs.* Week 13 and Week 0 *vs.* Week 26).

Linear multiple regression analysis was applied to evaluate the intervention effect on whole blood selenium and plasma SelP. The mean changes of whole blood selenium and plasma SelP from Week 0 to Week 13 and from Week 0 to Week 26 were plotted using a QQ-plot and found to be normally distributed. Within the two groups mean changes (Weeks 0–13, Weeks 0–26) and the difference between the groups’ mean changes (Weeks 0–26) were calculated from the linear multiple regression model using least squares means. The results are presented as mean change (95% CI).

To illustrate the range of variation in the groups at the three measurements (Weeks 0, 13, 26), a box plot with 5th, 25th, 50th, 75th and 95th percentiles is presented.

In a sub-analysis, we evaluated whether selenium supplement consumed between 3 and 12 months before study recruitment was an effect modifier on the intervention effect. An interaction term between selenium supplement consumption and intervention group was included in the model.

The procedure general linear model (GLM) in the Statistical Analysis Systems statistical software, release 9.3 (SAS Institute, Cary, NC, USA) was used for the analyses.

## 3. Results

### 3.1. Study Participants

**Figure 1:**
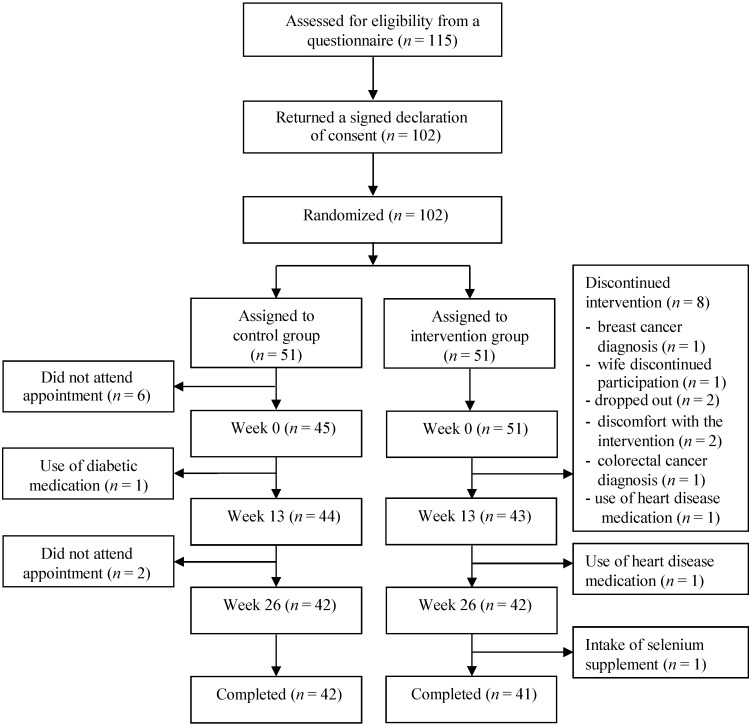
Flow diagram of the recruitment, randomization, and study flow.

Although 115 persons were assessed for eligibility, only 102 agreed to be enrolled and were subsequently randomized to the intervention or control group (Figure 1). Of the 102 participants assigned to a group, 83 completed the study. In the control group, six participants were excluded because they did not attend the initial appointment. Further, one participant was excluded after the initial appointment due to use of medication for diabetes, and two were excluded because they did not attend the third appointment. In the intervention group, eight participants were excluded after the initial appointment (three due to diagnosis of disease, two due to discomfort with the intervention, one participant dropped out because his wife was excluded, and two participants dropped out without further explanation). Further, two participants deviated from the protocol and were both excluded from the analysis; they were found not to meet eligibility criteria regarding use of medication for heart disease and dietary supplementation with selenium, respectively. They informed us about the deviations after the second appointment and at study end.

### 3.2. Baseline Characteristics

The intervention and control group were similar regarding sex, age, BMI, and blood selenium and SelP (as selenium concentration) at baseline (Table 1).

### 3.3. Compliance

All participants showed a high compliance with the intervention. The median of ingested proportion was 99% of the received fish and mussels, and the participant with the lowest compliance ingested 89%.

### 3.4. Whole Blood Selenium

**Figure 2:**
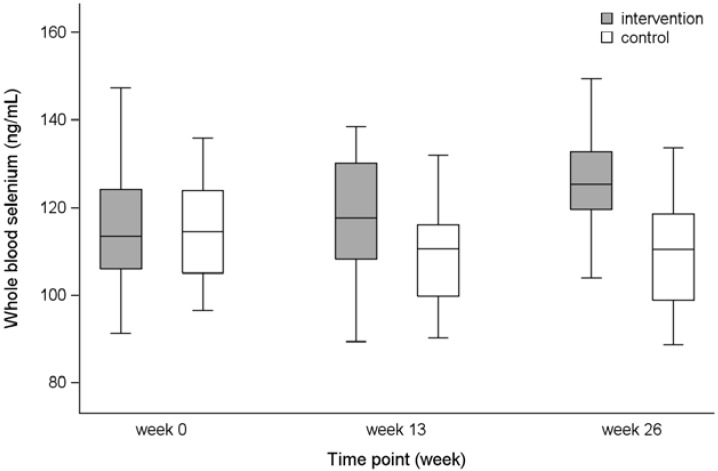
Whole blood selenium concentrations for the intervention and the control group presented as 5th, 25th, 50th, 75th and 95th percentiles for Weeks 0, 13, and 26.

The difference in mean change for intervention compared with control persons was 14.9 ng/mL (95% CI: 10.2, 19.7) for Weeks 0–26 (Table 2). In the intervention group the concentration increased, whereas a reduction occurred in the control group (Table 2 and Figure 2). It was studied whether use of dietary selenium supplement between 3 and 12 months before study recruitment modified the results (Table 3). Though tests for interaction gave *p*-values of borderline statistical significance (Weeks 0–13: *p* = 0.06, Weeks 0–26: *p* = 0.08), the pattern of the estimates was not biologically interpretable.

**Table 1:** Baseline characteristics presented as number (percentage) or median (5–95 percentiles).

Variable	Intervention (*n* = 49)	Control (*n* = 45)
Women	21 (43%)	19 (42%)
Men	28 (57%)	26 (58%)
Age, year	61 (51–72)	59 (51–73)
BMI, kg/m^2^	26.3 (21.0–31.1)	25.2 (20.3–32.5)
Whole blood selenium, ng/mL	113.5 (91.3–147.4)	114.6 (96.6–136.0) ^1^
Plasma selenoprotein P, ng selenium/mL	51.4 (35.0–64.0) ^2^	51.4 (35.4–65.7) ^3^
Plasma selenium, ng/mL	84.7 (67.8–106.5) ^2^	86.4 (70.5–103.3) ^3^

^1^ *n* = 44 due to exclusion of outlying values (*n* = 1), all whole blood selenium concentrations for this person were excluded; ^2^ *n* = 46 due to errors in the laboratory measures (*n* = 3), all plasma selenoprotein P concentrations and the plasma selenium concentration analyzed only at baseline were excluded for these persons; ^3^ *n* = 43 due to errors in the laboratory measures (*n* = 2); all plasma selenoprotein P concentrations and the plasma selenium concentration analyzed only at baseline were excluded for these persons.

**Table 2:** Change within group and difference between group changes in whole blood selenium and plasma selenoprotein P concentrations.

	Mean Change within Group (95% CI)				Difference between Group Mean Change (95% CI)	
	*n*	Weeks 0–13	*n*	Weeks 0–26	*n*	Weeks 0–26
Whole blood selenium, ng/mL						
Intervention	41	2.0 (−1.3, 5.2)	41	10.8 (7.4, 14.2)	82	14.9 (10.2, 19.7)
Control	43	−5.3 (−8.5, −2.1)	41	−4.1 (−7.5, −0.7)		
Plasma selenoprotein P, ng selenium/mL						
Intervention	37	1.9 (−1.2, 5.1)	38	10.2 (7.4, 13.0)	78	7.0 (3.1, 10.9)
Control	42	−5.6 (−8.5, −2.6)	40	3.2 (0.5, 5.9)		

**Table 3:** Change within group in whole blood selenium concentrations, stratified by intake of dietary selenium supplement between 3 and 12 months before study recruitment.

	Mean Change within Group (95% CI)				*P* Interaction	
	*n*	Weeks 0–13	*n*	Weeks 0–26	Weeks 0–13	Weeks 0–26
Intake of dietary selenium supplement					0.06	0.08
Intervention	19	−2.9 (−7.6, 1.7)	19	8.4 (3.5, 13.3)		
Control	18	−5.6 (−10.4, −0.9)	18	−1.9 (−6.9, 3.2)		
No intake of dietary selenium supplement						
Intervention	22	6.2 (1.9, 10.5)	22	12.9 (8.3, 17.5)		
Control	25	−5.1 (−9.1, −1.0)	23	−5.9 (−10.4, −1.4)		

### 3.5. Plasma SelP

**Figure 3:**
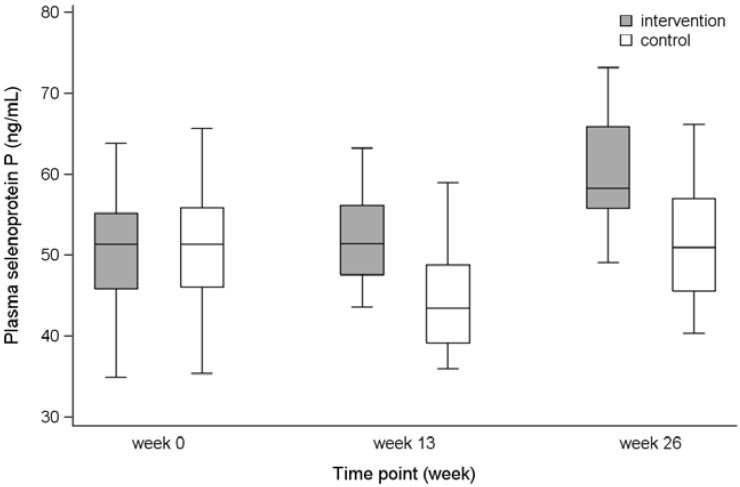
Plasma selenoprotein P concentrations for the intervention and the control group presented as 5th, 25th, 50th, 75th and 95th percentiles for Weeks 0, 13, and 26.

The difference in mean change for intervention compared with control persons was 7.0 ng/mL (95% CI: 3.1, 10.9) for Weeks 0–26 (Table 2). In the intervention group the concentration increased. In the control group, a reduction occurred in the first period (Weeks 0–13), but over the 26-week period the concentration increased (Table 2 and Figure 3). Dietary selenium supplement consumed before study recruitment was not an effect modifier on the intervention effect (Weeks 0–13: *p* = 0.87, Weeks 0–26: *p* = 0.49) (results not presented).

## 4. Discussion

This dietary intervention study included healthy middle-aged participants with low habitual dietary selenium intake. We found that a 26-week intervention with fish and mussels significantly increased the whole blood selenium and plasma SelP concentration in the intervention group when compared with a control group.

The main strength of our study is its design where the randomization procedure is expected to assure that known and unknown confounders are evenly distributed between the two groups. However, a larger sample size may have been required to rely completely on the randomization effect. The restrictive inclusion and exclusion criteria further reduce the potential influence of confounding, and the long-term duration of the intervention combined with the high compliance regarding the intervention diet is an additional strength. The high compliance indicates that the experiment was well designed inasmuch as the intervention group was able to comply with the intervention diet, albeit compliance was assessed based on self-monitored records only and therefore bias cannot be excluded. Intention-to-treat analysis is generally the favored method for randomized studies. However, in the present study, a few participants provided only the baseline blood sample or the two first blood samples (Weeks 0 and 13) and an intention-to-treat analysis would have required imputation of the missing data for these persons. Imputation of missing data could introduce problems, especially in smaller studies such as the present one. We therefore based our data analyses on available observations only and this should be taken into account when considering the results. Due to the chosen methods of data analysis, we are not able to evaluate the intervention effect in the group of participants that dropped out. The dropout in the present study was not higher than expected (around 20%) and was almost the same in both groups (Figure 1). Thus, it seems unlikely that the study dropout was related to the intervention. Further, it is not likely that the dropout was related to the effect of the intervention (Figure 1). Selection bias is therefore not expected to have influenced the results.

Due to the intervention with a high amount of fish, it was not possible to blind the participants to intervention or control status. We would expect the lack of blinding primarily to lead to a higher intake of fish in the control group. This is not indicated by the results, as blood selenium concentrations in the control group did not increase (see Table 2).

Our participants were non-fasting at blood sampling, and the biological variability of selenium status is another aspect that needs to be considered. The biological half-life of selenium in blood plasma is relatively short (approximately eight hours) [25]. Plasma selenium responds rapidly to short-term changes in dietary selenium intake [4] and time since last intervention meal may therefore influence the plasma selenium concentration at blood sampling. To account for this, we used whole blood selenium as our primary outcome measure as this marker has been shown to reflect long-term selenium status [26,27]. Plasma selenium was estimated only for baseline samples to enable comparison with other Danish studies measuring plasma selenium in a healthy population.

The intervention was conducted over 26 weeks, as this was expected to be sufficiently long to maximize SelP concentration. Previous studies investigating SelP concentration suggest that the maximal plasma SelP concentration is reached before 10 weeks of supplementation [5,28]. However, a study measuring whole blood selenium indicated response of supplementation up to 35 weeks but with a flattening of the curve after approximately 20 weeks [29]. We would therefore expect any potential effect beyond the 26 weeks of intervention in the present study to be modest and, consequently, that the duration of our intervention was sufficient.

Few intervention studies have investigated the effect of fish and shellfish intake on selenium concentrations, and the results are inconsistent. In a population from New Zealand (*n* = 11) with baseline plasma selenium concentrations around 120 ng/mL, an intervention with 240 g of salmon/week for eight weeks (~47 μg selenium/week) was compared with a control group consuming salmon oil capsules [21]. The study found that plasma selenium increased significantly in the fish-eating group when compared with the group receiving capsules (mean difference around 11 ng/mL). However, in a Norwegian study (*n* = 11) with similar baseline selenium concentrations, a considerably higher intake of fish (1500 g of different fish/week for six weeks ~420 μg selenium/week) did not result in a significant increase in plasma selenium [17]. In our study, the baseline concentration of plasma selenium was around 85 ng/mL. We found that a 26-week intervention with 1000 g of fish and mussels/week (~350 μg selenium/week) significantly increased the whole blood selenium concentrations (mean difference around 15 ng/mL), which falls within the range of observations reported previously.

Baseline concentrations of plasma selenium were estimated to allow comparison with plasma selenium concentrations among healthy participants in other Danish studies. In our study the median concentrations were 85 ng/mL in the intervention group and 86 ng/mL in the control group at baseline. These concentrations were slightly lower than in another Danish study finding plasma selenium concentrations of 93 ng/mL in healthy, middle-aged participants (*n* = 24) [7]. Whole blood selenium at baseline in the present study (around 114 ng/mL) was similar to levels found in other Danish studies in healthy participants (range: 95–120 ng/mL) [30,31,32].

SelP is the major plasma selenoprotein and contains at least 50% of all selenium in plasma [33]. In the present study, we found that SelP accounted for 59.5% (median) of total plasma selenium at baseline (5–95 percentiles, 49.7%–65.6%), which is in line with previous findings.

According to our study protocol, we expected a mean whole blood selenium concentration around 155 ng/mL at study end in the intervention group corresponding to a plasma concentration of approximately 130 ng/mL. This approximation was based on a Danish pilot study (unpublished results) in which plasma and whole blood selenium were measured following intervention with selenium-enriched yeast. Nevertheless, the intervention group in the present fish intervention study did not reach the expected whole blood selenium concentration (median at study end was 125 ng/mL, see Figure 2) and saturation of SelP was therefore not achieved [5]. The modest increase in selenium could be due to a decrease in intake of other selenium-containing foods among the participants in the intervention group; for example, they may have substituted some of their meat and seafood intake with the fish and mussels provided through the intervention. As meat is an important source of selenium in the Danish diet [20], such a substitution could result in a lower than expected selenium intake. The estimated selenium intake (~350 μg/week) was based on data on the concentration of selenium in food from the Danish Food Database [18]. The selenium content in fish and mussels may have varied during the 26-week intervention; accordingly, the selenium intake could have been lower or higher than that estimated. A possible modest bioavailability in humans of selenium from fish and shellfish (56%–88%) [14,15,16,17] could be an additional explanation for the lower blood selenium concentration at study end. Furthermore, *in vitro* studies of the bio-accessibility of selenium from seafood showed that a minor fraction of low-molecular selenium species was accessible [34]. This could imply that the issue of modest bioavailability of selenium from fish is associated with the digestibility of selenium contained in the protein fraction of the intervention diet. Finally, it has been suggested that mercury, a known contaminant present in fish, may interact with selenium and reduce its bioavailability [35,36,37].

During the first half of the study (Weeks 0–13) we found an unexpected decreasing concentration of selenium in the control group. The decrease in selenium concentrations in the control group from baseline to Week 13 and the potentially limited increase in selenium concentrations in the intervention group were unexpected and are difficult to explain. One reason could be a seasonal variation in general food intake or selenium content of specific foods that may influence the selenium intake. A few previous studies have investigated seasonal variation in blood selenium concentrations. In accordance with the present study, the lowest concentration of plasma selenium was found from October to December in an older British population [38]. However, studies in other populations, including one in Danes [39], do not support any seasonal variation in blood selenium concentrations [28,40,41].

To ensure that the measured blood concentrations of selenium were of dietary origin, use of supplements containing selenium three months before study baseline was an exclusion criteria. An additional explanation for the unexpected decreasing concentration in blood selenium during the first 13 weeks could be that this washout period was too short. Nevertheless, when looking into pre-study users and non-users of selenium-containing supplements we did not find a pattern indicating that the decrease in selenium blood concentrations among the control persons was more pronounced among pre-study supplement users (Table 3).

The concentration of SelP may not have reached its maximum when looking at the whole blood selenium concentrations reached from the intervention (expected concentration: 155 ng/mL, concentration at study end: 125 ng/mL). An even higher intake of selenium through the diet may accordingly be necessary to maximize SelP and thereby obtain the suggested preventive effect of selenium against chronic diseases. However, the actual associations between SelP concentrations and cancer incidence are unknown [42,43]. Genetic variations in selenoproteins have been suggested to influence the concentration of the selenoproteins and thereby the response to intake of selenium [44]. This is a relevant factor that may have influenced the individual SelP concentrations in our study population, but it was not evaluated in the present study.

The present intervention indicated that it is difficult to increase the selenium blood concentrations only by increasing the intake of selenium-rich foods, such as fish and mussels, particularly because the concern about potential contaminants in fish must also be taken into account. Other potential strategies for increasing the selenium intake and the subsequent selenium blood concentrations are, for example, agronomic biofortification by adding selenium to fertilizers as a public-health solution or through intake of dietary selenium supplements [12]. In Finland the former strategy has increased the plasma selenium concentration from 70 ng/mL to 110 ng/mL in the healthy adult population [13]. Supplementation with selenium has been evaluated in several studies and seems to be an effective way to increase blood levels of selenium [26]. A long-term supplementation study has previously been conducted in a healthy Danish study population showing that the mean plasma concentration was increased to 165 ng/mL when a supplement with selenium-enriched yeast (100 μg/day) was given [7]. The selenium status suggested as optimal for human health according to prevention of certain cancers is within a narrow range [2], which may complicate the use of these strategies at population level.

## 5. Conclusions

In conclusion, we found that an increased intake of fish and mussels significantly increased the whole blood selenium and plasma SelP concentration in a population with relatively low habitual dietary selenium intake. However, the increase in selenium concentrations in the intervention group was lower than expected.
